# Efficacy of Physiotherapy Rehabilitation for Subtrochanteric Femur Fracture: A Case Report

**DOI:** 10.7759/cureus.50822

**Published:** 2023-12-20

**Authors:** Devashish Thote, Shraddha S Kochar, Maithili M Deshpande, Ritik V Daf

**Affiliations:** 1 Musculoskeletal Physiotherapy, Ravi Nair Physiotherapy College, Datta Meghe Institute of Higher Education and Research, Wardha, IND

**Keywords:** post-fracture surgery, plate osteosynthesis, pain, physiotherapy, subtrochanteric fracture, comprehensive rehabilitation

## Abstract

Proximal femur fractures that occur within 5 cm of the lesser trochanter are commonly referred to as subtrochanteric femur fractures (STF). In this case report, we depicted a 45-year-old who came with a history of road traffic accident (RTA) for which an investigation like an X-ray was performed which revealed STF and the patient was surgically managed. Postoperatively, the patient's main complaints were pain around the hip joint with restriction in performing hip joint full range of motion. For these complaints, she was given physiotherapy. There was a reduction in joint pain, a significant improvement in joint movement, and increased muscle strength which was observed after evaluating the outcome measures.

## Introduction

Femur fractures that occur proximally less than 5 cm from the lesser trochanter are referred to as subtrochanteric femur fractures (STF). According to calculations, there are 15-20 of these kinds of fractures for every 100,000 individuals, and there are bimodal age distributions for these fractures, which consist of 20% of STF occurring in individuals under 40 and more than two-thirds occurring in those over 50 [1]. At earlier ages, there is a nearly equal occurrence of these fractures in males and females. However, as people get older, there are more chances of occurrence of this fracture [1]. Early mobilization and preventing the effects of prolonged immobility are crucial because of the high occurrence of STF in older persons. It is more commonly seen in females because of early degenerative changes due to menopause. These fractures are more difficult for the treating physician because of the unusuality of the subtrochanteric site. Fracture reduction is difficult because of deforming muscle forces, strong tensile strains laterally, and compressive stresses medially. Additionally, the subtrochanteric region's blood supply is more susceptible [2].

The anatomical site of the fracture must be clearly defined when analyzing a specific bony injury. After identifying the location of the affected bone, the subgroups within the fracture can be further divided. The ideal categorization system should be useful in deciding the selection of treatment and prognosis and have minimal differences between and among observers [3]. Anatomical reduction and surgical fixation serve as the foundation of the therapy of STF. Rarely, non-surgical treatment is only used for the sickest patients with the most severe comorbidities, in whom a surgical procedure would directly endanger their lives. However, in these situations, the frequency of difficulties with fracture healing and prolonged immobilization is associated with poor results [4]. Until now, intramedullary nails, angled blade plates, locking plates, dynamic hip screws (DHS), dynamic condylar screws (DCS), and dynamic condylar plates have all been utilized to fixate STF definitively. Despite trials demonstrating each of these divisions' effectiveness, the DHS and DCS have a higher chance of reduction loss, implant failure, and the necessity for general reoperation [5-7].

With intramedullary fixation, STF can be treated. The clinical result and the prognosis of the patients depend on the precision of intraoperative reduction and surgical expertise [6]. Many instruments can aid in lowering the incidence of STF when paired with steady traction and an altered entry site [7]. According to the available data, the primary factor determining the outcome of STF is the quality of reduction alone [8]. The intramedullary fixation devices provide the most consistent results in terms of stable fixation, maintenance, and biomechanical superiority [9]. The 45-year-old female patient in this case study had an STF who needs efficient physical therapy after surgery to lower the possibility of problems, and internal fixation and open reduction were performed. Previous studies for the care of such fractures have only presented a limited number of physical therapy modalities and have not provided a thorough week-by-week rehabilitation strategy. A weekly treatment plan that includes physical therapy techniques was created in response to this need and is used in the treatment of STF.

## Case presentation

Patient information

A 45-year-old female who is a housewife came with a history of road traffic accident (RTA) while she was crossing the street and was struck by a vehicle. On August 8, 2023, the patient was taken to a government hospital with complaints of pain, which began suddenly and was severe. The pain was aggravated by the movement of the left limb and alleviated after taking medicine and relaxation. The patient was unable to move or stand up. She underwent investigation like an X-ray and was diagnosed with an STF of the left side where primary treatment was given with a pelvic binder. She gives neither a history of any comorbidities nor bladder or bowel complaints. No other previous illness or surgical history was found. Afterward, the patient visited Acharya Vinoba Bhave Rural Hospital (AVBRH) where she was suggested for surgery on STF of the left side. After the surgical procedure, the patient's main complaint was pain around the hip joint and restrictions in the hip joint movement; therefore, for additional care, the patient was sent to musculoskeletal physical therapy. Table 1 shows the timeline of the patient.

**Table 1:** Timeline of the patient

Events	Dates
Date of admission	10/08/2023
Date of surgery	19/08/2023
Date of physiotherapy rehabilitation	23/08/2023
Date of discharge	25/10/2023
Date of follow-up	31/10/2023

Clinical findings

Before the commencement of the examination, informed consent was taken from the patient, and she underwent an examination. She appeared to be cooperative and well aware of the time, location, and people around her. The patient was hemodynamically stable. With the lower limb supported by a cushion underneath, the patient was observed in a supine lying position. She was mesomorphic in built with a body mass index (BMI) of 22 kg/m^2^. The swelling was presented around the left hip. The left leg was externally rotated. Palpation revealed grade 2 tenderness (the patient complains of pain and winces) in the left leg's proximal thigh area. There was a little increase in local temperature. Upon assessment, the left leg was found to have quadriceps muscle wasting. On a Numerical Pain Rating Scale (NPRS), the pain was evaluated at 9/10 during movement and 5/10 during rest.

Investigatory findings

**Figure 1:**
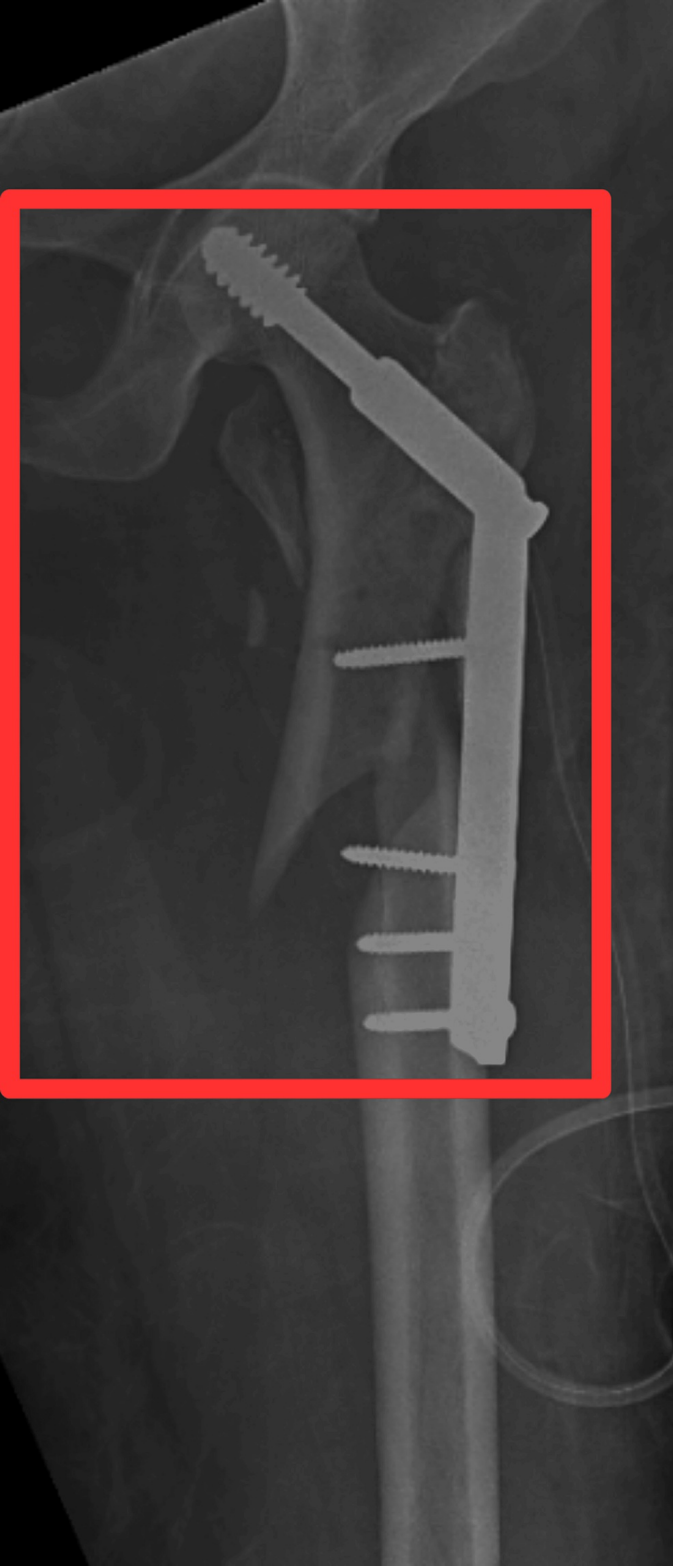
X-ray showing the surgical correction of the subtrochanteric fracture of the left side The red rectangle shows the open reduction and internal fixation done with the help of screws and plates

Figure 1 shows the postoperative X-ray of the patient. 

Physiotherapy interventions

**Figure 2:**
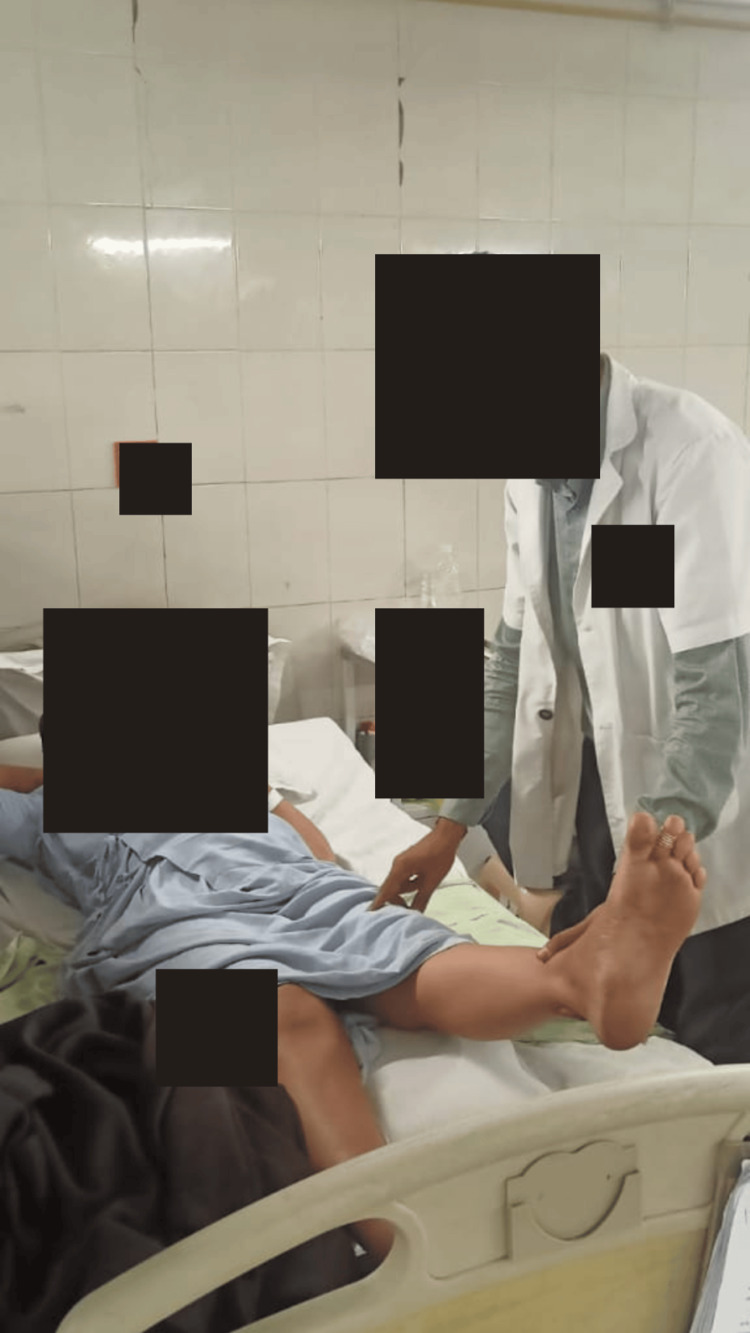
Patient performing straight leg raise with minimal support

**Figure 3:**
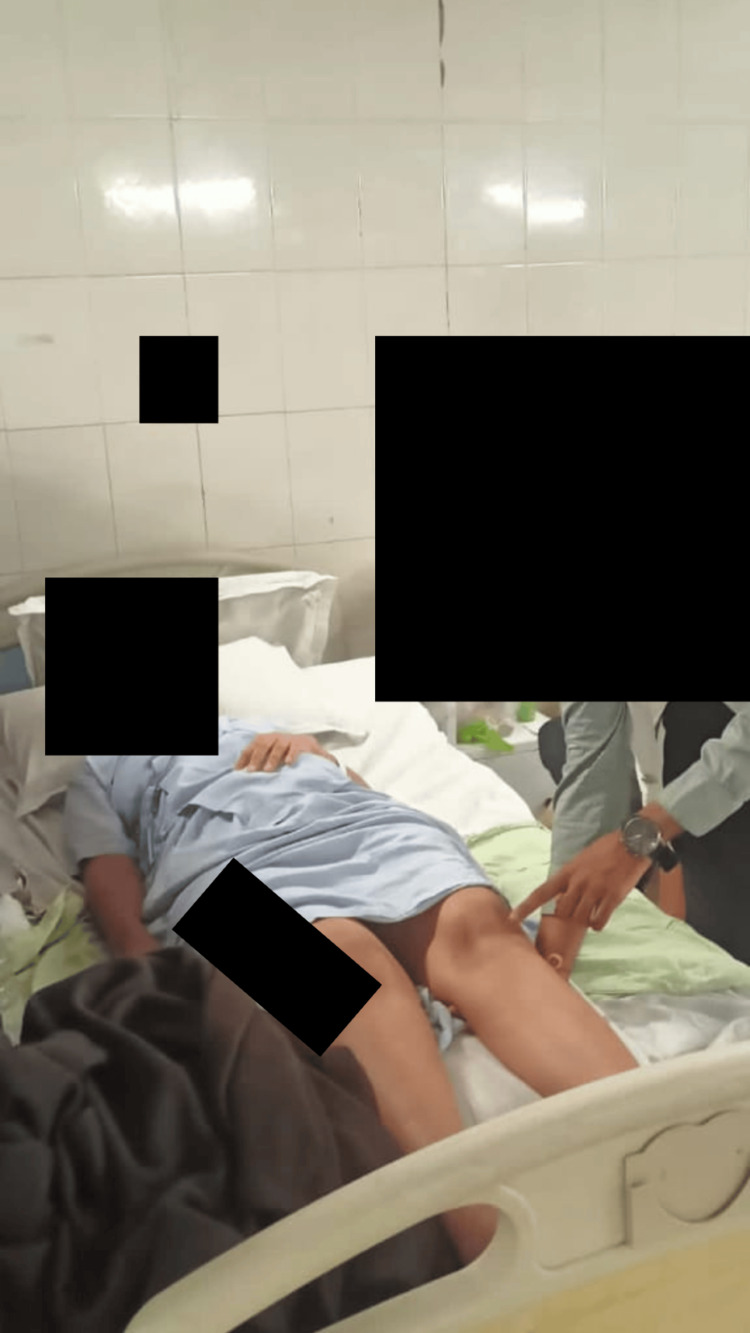
Patient performing static quadriceps

The aim of the patient's rehabilitation was to return her to normal activities of daily living (ADLs) as much possible. For eight weeks, the patient underwent physiotherapy treatment. The patient's physiotherapy care from week 1 to week 8 is shown in Table 2. Figure 2 and Figure 3 show the patient being rehabilitated.

**Table 2:** Physiotherapy management reps: repetitions; SLR: straight leg raise: ADLs: activities of daily living; °: degree

Weeks	Goals	Therapeutics intervention
Weeks 1-4	To lessen pain at the fracture site	Ice therapy, 10 minutes three to four times daily
	To improve the motion of the knee and hip joints	Active range of motion of the knee and hip when lying supine, 10 reps x 1 set from week 1 progressing to 20 reps for week 4
	To strengthen the muscles around the knee and hip joints	Isometric exercises for the hamstring, gluteal, and quadriceps. Static quadriceps and hamstring exercises, ankle isotonic exercises, SLR, 10 reps x 1 set from week 1 progressing to 20 reps for week 4
	Starting to ambulate and bear weight on the injured leg	Exercises prior to bearing weight which were given were knee walking, four-point kneeling, and prone lying (week 1), toe touch partial weight bearing (week 2), and ambulation using stand pivot transfers and walkers as mobility aids (weeks 2-4)
	To enhance and adapt the ADLs	Using both the chair and an elevated toilet seat, rolling to the unaffected side before getting out of bed, and doing so for two to four weeks
Weeks 5-8	To enhance and preserve the range of motion of the knee and hip	Self-assisted heel slides beyond 90^o^, 10 reps x 1 set progressing to 20 reps
	To improve the endurance of the muscles around the knee and hip joints	Exercises involving resistance for the hip and knee muscles, leg and gluteus medius isometric exercises, 10 reps x 1 set progressing to 20 reps
	To enhance the patient's ability to bear weight and walk	Walking and transferring with the use of assistive technologies; three-point gait pattern; a three-point gait pattern was started with the use of crutches from week 5 onwards
	To enhance and adapt the ADLs	Independent movement in bed; apart in terms of attire, the patient starts to become independent after week 8

Follow-up and outcome measures

At the end of the eight weeks of rehabilitation, the patient might execute all ADLs and doesn't complain of any pain or discomfort. She was satisfied with the prescribed protocol and was capable of doing everyday tasks comfortably which ultimately improved her quality of life (QOL). Table 3 and Table 4 show the results of range of motion (ROM) and manual muscle testing (MMT) prior to and after therapy.

**Table 3:** Pre- and post-rehabilitation ROM ROM: range of motion

Parameter	Pre rehabilitation		Post rehabilitation	
Left hip ROM (in degree)	Active	Passive	Active	Passive
Flexion	0-40	0-45	0-100	0-105
Extension	0-10	0-15	0-15	0-20
Abduction	0-10	0-15	0-15	0-20
Adduction	15-0	18-0	15-0	20-0
Internal rotation	0-10	0-15	0-15	0-20
External rotation	0-5	0-10	0-15	0-20

**Table 4:** MMT MMT: manual muscle testing 0: No visible or palpable contraction. 1: Flickering of contraction. 2: Full range of motion with gravity eliminated. 3: Full range of motion with against gravity. 4: Full range of motion with against gravity with minimal resistance. 5: Full range of motion with against gravity with maximal resistance

MMT (left hip)	Pre treatment	Post treatment
Hip flexors	2/5	4/5
Extensors	2/5	4/5
Abductors	2/5	4/5
Adductors	2/5	4/5

## Discussion

As said by Lalwani et al., physiotherapy plays a very crucial role in the rehabilitation of the patient with STF. Pain intensity was reduced by using cryotherapy, ankle toe movement was given to prevent edema, ROM exercises were implemented to regain joint mobility, and early mobilization was initiated to enhance the patient's QOL [10]. This rehabilitation protocol was also implemented on the patient, and positive results were observed.

Stabilizing the fracture to permit at least 50% weight bearing through the leg and re-establishing limb alignment are the primary objectives of surgery [11]. The prognosis of pelvic traumas with bleeding depends on quick treatment [12]. Rehab objectives were established, starting with simple exercises to weight bearing ambulation with a walker and strengthening [13]. Appropriate weight bearing status should be prescribed by the medical or surgical team; following these guidelines is essential for the best possible recovery because early weight bearing might impede the healing process [14].

To prevent the fracture from happening again, the patient got up from the floor, refused to sit with his legs crossed, and kept both legs apart. These therapy strategies improved the patient's well-being and outcomes [15]. Trauma patients who receive more intense acute inpatient physical therapy are more likely to return home, and new research has shown that this relationship is reliable, leading to quicker mobility improvements [16]. The demand for assistance equipment and the duration of the patient's stay at an assisted living facility increase after surgery, making the rehabilitation process more difficult [16]. According to Gabriel's study, postoperative physiotherapy had a good effect on improving gait and boosting self-confidence. For postoperative patients to heal, a structural rehabilitation program is always recommended according to their functional requirements and physical state to obtain favorable prognostic outcomes [17,18].

A skilled musculoskeletal physiotherapist provided the patient with a comprehensive physical therapy rehabilitation program including a variety of exercises and equipment for resistance, according to the most recent report. Significant pain relief with cryotherapy and painkillers allowed the patient to focus more on their rehabilitation, which improved their joint mobility incorporating weight training in addition to their clinical condition [19,20]. The goal of the physical therapy sessions was to maintain the integrity of the muscles in the right lower leg while simultaneously improving the function of the left lower limb and both upper limbs. This would enable each patient to walk independently with the use of an aiding mechanism and little assistance for daily tasks. The individual receiving treatment was given a recorded regimen, instructed to complete the majority of the exercise as a component of a home regimen, and asked to return for follow-up appointments.

## Conclusions

The program for physical and mental well-being after a fracture is effective, showing notable gains in both areas. The case study provides a detailed plan of care for individuals who have received post-fracture surgery. Most of the therapeutic goals had been met, including enhanced strength of the muscles, gradually expanding hip ROM, enhanced functionality, decreased pain, and enhancements in the patient's gait and everyday activities after the completion of centered physical therapy.
